# A scoping review of statistical methods in studies of biomarker-related treatment heterogeneity for breast cancer

**DOI:** 10.1186/s12874-023-01982-w

**Published:** 2023-06-29

**Authors:** L Sollfrank, SC Linn, M Hauptmann, K Jóźwiak

**Affiliations:** 1 Institute of Biostatistics and Registry Research, Brandenburg Medical School Theodor Fontane, Fehrbelliner Straße 39, Neuruppin, 16816 Germany; 2 Division of Molecular Pathology, The Netherlands Cancer Institute, Amsterdam, The Netherlands; 3 Department of Medical Oncology, The Netherlands Cancer Institute, Amsterdam, The Netherlands; 4 Department of Pathology, University Medical Center, Utrecht, The Netherlands

**Keywords:** Predictive, Biomarker, Treatment heterogeneity, Interaction, Breast cancer, Review, Statistical methods

## Abstract

**Background:**

Many scientific papers are published each year and substantial resources are spent to develop biomarker-based tests for precision oncology. However, only a handful of tests is currently used in daily clinical practice, since development is challenging. In this situation, the application of adequate statistical methods is essential, but little is known about the scope of methods used.

**Methods:**

A PubMed search identified clinical studies among women with breast cancer comparing at least two different treatment groups, one of which chemotherapy or endocrine treatment, by levels of at least one biomarker. Studies presenting original data published in 2019 in one of 15 selected journals were eligible for this review. Clinical and statistical characteristics were extracted by three reviewers and a selection of characteristics for each study was reported.

**Results:**

Of 164 studies identified by the query, 31 were eligible. Over 70 different biomarkers were evaluated. Twenty-two studies (71%) evaluated multiplicative interaction between treatment and biomarker. Twenty-eight studies (90%) evaluated either the treatment effect in biomarker subgroups or the biomarker effect in treatment subgroups. Eight studies (26%) reported results for one predictive biomarker analysis, while the majority performed multiple evaluations, either for several biomarkers, outcomes and/or subpopulations. Twenty-one studies (68%) claimed to have found significant differences in treatment effects by biomarker level. Fourteen studies (45%) mentioned that the study was not designed to evaluate treatment effect heterogeneity.

**Conclusions:**

Most studies evaluated treatment heterogeneity via separate analyses of biomarker-specific treatment effects and/or multiplicative interaction analysis. There is a need for the application of more efficient statistical methods to evaluate treatment heterogeneity in clinical studies.

**Supplementary Information:**

The online version contains supplementary material available at 10.1186/s12874-023-01982-w.

## Introduction

Breast cancer (BC) is the most common cancer among women worldwide [1]. In Europe, one in 11 women is diagnosed with BC at least once in her life [2]. Although 5-year survival rates after BC diagnosis increased steadily during the last decades and currently exceed 80% [2], modern treatments still fail in many patients causing significant morbidity and mortality.

At the same time, thousands of scientific papers are published annually and substantial resources are spent to develop tests for precision medicine in oncology. These tests are usually based on biomarkers, e.g., characteristics measurable in healthy or tumor tissue which influence a patient’s clinical outcome. Prognostic biomarkers describe the likelihood of a future recurrence or progression of cancer, i.e., they identify patients who require additional systemic therapy besides local therapy like surgery or radiotherapy - they indicate who needs additional therapy. Predictive biomarkers identify patients who are more likely to respond to a treatment, i.e., they select the most promising treatment for a specific patient - they indicate how one should be treated [3]. Therefore, predictive biomarkers are essential for personalized medicine and are a topic of current research, e.g., mutations in the BRCA1 gene [4, 5] or tumor infiltrating lymphocytes (TILs) [6, 7]. However, only a handful of these tests are currently being used in clinical practice, for example, presence/absence of human epidermal growth factor 2 (HER2) gene amplification or estrogen receptor (ER) in BC [8, 9]. The reason is that a candidate biomarker has to pass several stages of development. Perhaps the most challenging stage is the translation from a convincing preclinical test to using the same test in patients with cancer in daily practice. To demonstrate clinical utility of a test, series of patients are required who received the treatment of interest or an alternative treatment and have positive or negative test results.

Obtaining conclusive results in such studies depends on the choice of the study design and the statistical method for data analysis. Several guidelines for designing biomarker studies are available [10–14]. A commonly used statistical technique to evaluate a predictive biomarker is a test for interaction between biomarker and treatment in a cohort of suitable patients. This approach aims to evaluate, whether a relative benefit of a specific experimental treatment compared with a control treatment differs by biomarker level. An example of such a test is a comparison of the benefit of adjuvant tamoxifen versus no tamoxifen on the risk of BC recurrence between ER positive and negative disease [15]. In this context, there is an important distinction for clinical utility between “quantitative” and “qualitative” interaction. If a new treatment benefits all patients relative to standard, but a biomarker only associates with magnitude (“quantitative” interaction) but not direction of the effect (“qualitative” interaction), then the predictive biomarker is not likely to alter therapy choice if both effect sizes are clinically meaningful, and hence the marker is not clinically useful [16]. In statistical terms, the interaction analysis evaluates departure from a multiplicative model for the joint relative effect of biomarker and treatment on the outcome. However, interaction analyses are known to require large series of patients [17, 18], which may not be available, or performing many measurements may be too expensive.

The spectrum of statistical methods commonly used in studies evaluating biomarkers for BC treatment heterogeneity is unknown. Such knowledge, however, is essential to determine whether developing or using alternative statistical methods offers an opportunity to advance precision medicine for BC. We therefore provide a methods review of a representative sample of observational and randomized studies on predictive biomarkers for BC. We focus on study designs, statistical methods and sample sizes.

## Methods

The study complied with reporting recommendations for scoping reviews (PRISMA-ScR) criteria [19], although a protocol does not exist.

### Eligibility criteria

Eligible for our review were studies among female patients with BC comparing at least two different treatment groups, one of which chemotherapy or endocrine treatment, by levels of at least one biomarker. Reviews and other reports without original data were ineligible.

### Search strategy

A query was developed for a literature search of publications written in English and available in PubMed (Additional File 1). The query was then limited to 2019 as year of publication and to the following 15 journals:

*Annals of Oncology, Breast Cancer Research, Breast Cancer Research and Treatment, Clinical Cancer Research, International Journal of Cancer, Journal of the American Medical Association*, *Journal of the American Medical Association Oncology, Journal of Clinical Oncology, Journal of the National Cancer Institute, The Lancet, Lancet Oncology, Molecular Cancer Therapeutics*, *Nature Medicine, New England Journal of Medicine* and *PloS One.*

Full text versions of all identified articles were obtained. Based on a title and abstract review by at least two authors (LS, MH, KJ), ineligible articles were identified and excluded. Supplements, if any, were obtained for eligible articles.

### Data extraction

For eligible articles, we abstracted the following information: first author, journal, type of study, patient inclusion criteria, endpoint definition(s), biomarker(s) analyzed, experimental and standard treatment, total number of patients included in the original study and in the analyses of treatment effects by biomarker level, number of patients and events by biomarker level and treatment, median follow-up time, details about the statistical analysis and results.

Abstraction was done independently by at least two authors (LS, MH, KJ). Discordance was resolved by consensus. Extracted data were checked by three authors (LS, MH, KJ) for completeness. Extracted data were summarized in Additional File 2: Supplementary Tables 1–3.

Since several studies reported results for various biomarkers and/or endpoints, the description in this report was a selection. We presented results for up to two biomarkers reported in the article abstracts. If the abstract mentioned more than two biomarkers, we chose the two which appeared most important based on the amount of details provided and the strengths of the results. We omitted biomarkers that were only reported in the articles’ supplements. We summarized results in the total patient group and up to one subgroup mentioned in the abstract. If different types of endpoints were reported (binary and survival endpoints), we described them separately. We reported up to two time-to-event endpoints. We reported hazard ratios (HR) and odds ratios (OR) for effects of treatment, biomarker and/or interaction of both, as well as p-values for the interaction coefficients. For significant interaction tests, we reported whether the interaction was quantitative (i.e., the treatment effects differed in magnitude but not direction between marker levels or the effects of a continuous marker differed in magnitude but not direction between treatment groups) or qualitative (i.e., the treatment effects differed in direction between marker levels or the effects of a continuous marker differed in direction between treatment groups).

## Results

**Fig. 1:**
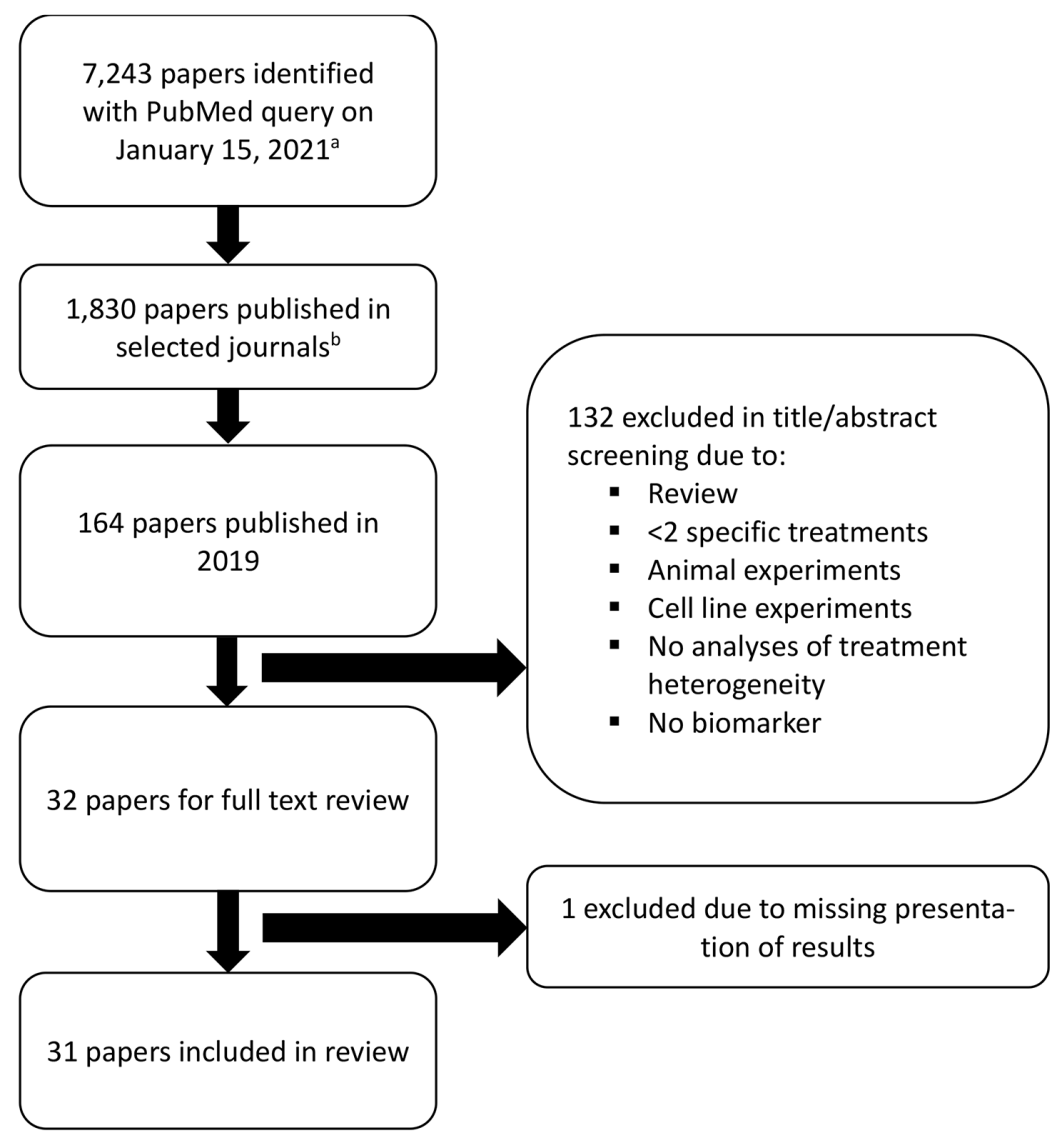
Flow chart of selection of relevant articles for the review ^a^ (“Breast Neoplasms”[Majr] OR ((breast[tiab] OR mammary[tiab]) AND (neoplas*[tiab] OR cancer*[tiab] OR tumor*[tiab] OR malignan*[tiab] OR oncolog*[tiab]))) AND (heterogeneity[TIAB] OR effect[TIAB] OR predict*[TIAB] OR prognostic[TIAB] OR interaction[TIAB]) AND (marker* OR biomarker*) AND (cohort[TIAB] OR patient*[TIAB] OR female[TIAB] OR women[TIAB]) AND (endocrine OR chemotherapy OR neoadjuvant) ^b^ Ann Oncol, Breast Cancer Res, Breast Cancer Res Treat, Clin Cancer Res, Int J Cancer, JAMA, JAMA Oncol, J Clin Oncol, J Natl Cancer Inst, Lancet, Lancet Oncol, Mol Can Ther, Nat Med, N Engl J Med, PloS one

The query performed on January 15, 2021, identified 7,243 articles of which 1,830 were published in the selected 15 journals. Of those, 164 articles were published in 2019 in 10 of the 15 journals (none published in the *Journal of the American Medical Association, The Lancet, Lancet Oncology, Nature Medicine* and *New England Journal of Medicine*). We excluded 132 of the 164 articles during title and abstract review and one during full text review (interaction described, but no results presented [20]), leaving 31 articles for description in this report (Fig. 1). These 31 articles were published in 6 of the selected journals, with 11 (35%) published in *Breast Cancer Research and Treatment*. Sample sizes ranged from 42 to 3,746 patients. One half of the studies included less than 367 patients, while one fourth included less than 175 patients. On average, these analyses included 52% of the original sample size. Median follow-up time, if reported, was mostly between 5 and 10 years. Most studies (23 of 31 (74%)) used patient data from randomized controlled trials and 20 (87%) of these studies used archived specimens. Time-to-event endpoints (progression or mortality) were evaluated in 25 of the 31 studies (81%), 7 studies (23%) evaluated binary endpoints (usually pathologic complete response, pCR). About half of the studies evaluated 1 biomarker, while 7 (23%) studies evaluated 5 or more with a maximum of 18 biomarkers in one study [21]. Similarly, while most studies evaluated treatment heterogeneity for only one endpoint, about one third used two or more (Table 1, Additional File 2: Supplementary Table 3).

### Clinical characteristics

Studies were diverse in terms of types of biomarkers, treatments and patient selection. In total, over 70 different biomarkers were evaluated. Biomarkers described in at least two articles were age, ER, progesterone receptor status (PR), HER2, stromal TILs (sTILs) and PIK3CA. Biomarkers were often used in different parametrizations, i.e., continuously and categorically, or with different categorizations.

Tamoxifen treatment was investigated in 6 studies and was compared to either a treatment with an aromatase inhibitor or no/less tamoxifen. Four studies reported on the effect of adjuvant chemotherapy (ACT) in comparison to no ACT. Three studies compared trastuzumab with no trastuzumab and two studies described the AKT inhibitor and anti-HER2 tyrosine kinase inhibitors. Treatments in all other studies were unique. Patient selection was based on the tumor, node, metastasis (TNM) staging system in 23 of 31 studies. Twenty-six studies used hormone receptor status (HoR) and 6 selected patients based on age. Moreover, 9 studies used only one criterion for patient selection, while the remaining 22 studies used specific combinations of patients and tumor characteristics.

**Table 1:** Characteristics of the study design of selected studies of biomarker-based treatment heterogeneity

Characteristic	Number (%^a^)
Journal^b^	
Ann Oncol	4 (13)
Breast Cancer Res	3 (10)
Breast Cancer Res Treat	11 (35)
Clin Cancer Res	3 (10)
Int J Cancer	5 (16)
J Clin Oncol	5 (16)
Number of patients included in analyses of treatment heterogeneity	
< 100	3 (10)
100–300	10 (32)
300–500	4 (13)
> 500	14 (45)
Minimum	42
25% quantile	175
Mean (SE)	851 (192)
Median	367
75% quantile	858
Maximum	3746
Type of study	
Randomized	23 (74)
Observational	8 (26)
Endpoints for which treatment heterogeneity was evaluated^c^	
Binary	7 (23)
pCR	6 (19)
> 50% relative decrease in 11-gene proliferation signature	1 (3)
Time to event	25 (81)
Time to progression^d^	25 (81)
Time to death^e^	9 (29)
Number of biomarkers for which treatment heterogeneity was evaluated	
1	14 (45)
2–4	10 (32)
5–10	5 (16)
> 10	2 (7)
Number of endpoints for which treatment heterogeneity was evaluated	
1	21 (68)
2	7 (23)
3	3 (10)
Median follow up time	
≤ 5 years	4 (13)
5–10 years	9 (29)
> 10 years	2 (6)
Not applicable	6 (19)
Not reported	10 (32)

^a^ Percentage do not sum up to 100, if a study qualified for several categories or due to rounding

^b^ Journals with no relevant papers were omitted

^c^ One study analyzed binary as well as survival endpoints

^d^ Time to progression includes: BCFI: invasive breast cancer-free interval; BCFS: breast cancer-free survival, DDFS: distant-disease-free survival; DFS: disease-free survival; DRFI: distant recurrence-free interval; EFS: event-free survival; iDFS: invasive disease-free survival; MRFS: metastatic recurrence-free survival; PFS: progression-free survival; RFI: recurrence-free interval; RFS: recurrence-free survival

^e^ Time to death includes: BCSS: breast cancer-specific survival, OS: overall survival

### Statistical characteristics

The majority of articles used one of two common statistical approaches to evaluate heterogeneity of treatment outcome by biomarker level. The first approach, evaluating the significance of a multiplicative interaction term between treatment and biomarker in a regression model with individual terms for treatment and biomarker, was used in 21 studies (Table 2). In 15 of these studies (68%), only a Cox proportional hazards model for a time-to-event outcome was applied, in 5 studies (23%) only a logistic regression model for a binary outcome was used and 1 study presented results from interaction analyses with binary as well as time-to-event endpoints [21–41]. The second common approach, subgroup analysis of relative treatment effects by biomarker levels, was additionally used in 12 studies with time-to-event endpoints and 4 studies with binary endpoints. Two articles with time-to-event endpoints presented interaction analyses and relative biomarker effects by treatment subgroups. One study performed interaction analyses and both types of subgroup analyses for time-to-event as well as binary endpoints [30]. Eight studies showed only results from subgroup analyses: 3 evaluated the treatment effect by biomarker subgroups [42–44], 4 the biomarker effect by treatment subgroups [46–49] and 1 only presented Kaplan-Meier curves for recurrence-free survival for all biomarker-treatment combinations [50]. Thus, there were 20, 7 and 1 studies that performed subgroup analysis for treatment effect by biomarker subgroup, biomarker effect by treatment subgroup and all biomarker-treatment subgroups, respectively (Table 2).

Two studies showed Subpopulation Treatment Effect Pattern Plots (STEPP) in addition to results from a standard multiplicative Cox proportional hazards model with interaction terms and subgroup analysis [31, 41]. STEPP is a graphical tool which divides patients into partially overlapping subpopulations based on subsequent values of a continuous biomarker. For each subpopulation, results such as the treatment-related HR, the cumulative incidence or the absolute risk difference are calculated and then plotted against the midpoints of the biomarker values in corresponding subpopulations. The plot can reveal biomarker values for which the experimental treatment is superior to the alternative treatment [51]. One of the two studies performing STEPP additionally included a statistical test to evaluate whether there was a change in the HR over the different biomarker intervals [41].

One article used a Bayesian covariate-adjusted logistic model [52]. The study evaluated the pCR after treatment with oral pan-AKT inhibitorMK-2206 in subgroups of patients defined by biomarkers HER2, HoR and MammaPrint status. Patients within each biomarker combination were randomly and adaptively assigned to MK-2206 or control arms. The treatment was considered superior in a subgroup of patients with a particular biomarker combination when it had ≥ 85% Bayesian predictive probability of success in a hypothetical phase III trial.

In addition to results from a Cox proportional hazards model with interaction term and subgroup analysis, one study presented differences in the cumulative incidence obtained with a Kaplan-Meier method at a particular time point between two treatment arms separately by biomarker subgroups to illustrate an absolute treatment effect [24].

While 8 of the 31 articles reported results for only one analysis with regard to a predictive biomarker, many studies performed multiple evaluations, mostly for several biomarkers, but also for several outcomes and subpopulations (Additional File 2: Supplementary Table 3). Five studies performed 10 or more analyses on the same patients [21, 30, 35, 40, 41]. The maximum number of analyses performed was 20 in a study with 18 different biomarkers [21]. Only 3 of the studies with more than one analysis adjusted their results for multiple testing [40, 41, 46]. The adjustment in all three studies was done by controlling the family-wise type 1 error rate via a Benjamini-Hochberg correction.

**Table 2:** Characteristics of statistical analysis of selected studies of biomarker-based treatment heterogeneity

Characteristic	Number (%^a^)
Type of analysis	
Cox model with biomarker-treatment interaction^b^	16 (52)
Logistic model with biomarker-treatment interaction^b^	6 (19)
Subgroup analysis^b^	
Treatment effect by biomarker subgroup	20 (65)
Biomarker effect by treatment subgroup	7 (23)
All biomarker-treatment subgroups	1 (3)
Subpopulation Treatment Effect Pattern Plot	2 (6)
Bayesian covariate-adjusted logistic model	1 (3)
Difference in cumulative incidence at one time point	1 (3)
Test of interaction	
Yes	22 (71)
No	9 (29)
At least one significant result	
Yes	21 (68)
No	10 (32)

^a^ Percentages do not sum up to 100, if a study qualified for several categories or due to rounding

^b^ One study presented interaction models for binary as well as time-to-event endpoints, and additionally biomarker effects by treatment subgroup and treatment effects by biomarker subgroup and is included in more than one category

### Results of individual studies

Of the 31 reviewed studies, 21 (68%) claimed to have found evidence that at least one evaluated biomarker was predictive, i.e., treatment effects differed by biomarker level (Table 2). This conclusion was based on a significant interaction test in 13 of those studies (of which 11 referred to a qualitative interaction), while 8 studies used significant p-values from subgroup analyses as evidence of treatment heterogeneity. The remaining 10 studies (32%) concluded that there was no evidence of treatment effect heterogeneity by biomarker levels. The number of significant results per study was generally low and did not strongly depend on the number of analyses conducted within the study (Spearman correlation coefficient = 0.34, p = 0.060; Additional File 2: Supplementary Table 3). Nearly half (14) of the articles mentioned that the study was not designed for the objective and was too small. Seven of these studies presented significant findings for at least one biomarker.

## Discussion

This review shows that there were numerous studies of predictive biomarkers evaluating treatment heterogeneity among patients with BC. Most of these studies described biomarker-specific treatment effects in subgroup analyses and/or performed interaction analyses in standard multiplicative models.

The evaluation of treatment heterogeneity is usually not a primary objective of randomized clinical trials. The trials are powered to evaluate main effects of treatment, and are usually underpowered to evaluate interactions, since the sample size required for interaction analyses with adequate power can be much higher than that for main effects. For example, Brookes et al. [18] showed that detection of an interaction with adequate statistical power may require a 4-fold sample size compared with that for evaluation of a main effect of the same magnitude. Moreover, the sample size of biomarker-based analyses is often even smaller than that of the primary analysis (in our review 48% smaller, on average) due to the failure to locate tissue samples, assay failure and quality control exclusions, when archived specimen are used. Besides sample size, statistical power depends on many factors: the baseline event rate (i.e., for the group of patients with low biomarker level and standard treatment), the proportions of patients by treatment and biomarker level, and either (i) the biomarker effect in one of the treatment subgroups, the treatment effect in one of the biomarker subgroups and the interaction effect, or (ii) the marginal effects of biomarker and treatment and the interaction effect. Methods and software tools are available to support the design of treatment heterogeneity analyses prior to the study [17, 53, 54]. It is unclear what the statistical power of the treatment heterogeneity analyses in the reviewed studies was when they were planned. It may not have been determined at all at that time since treatment heterogeneity was likely not the primary objective of most studies. Calculating post-hoc power based on observed data is meaningless [55]. Nevertheless, a test of interaction is required to rigorously assess whether treatment effects are different in biomarker subgroups [56, 57]. Evaluating only treatment effects in separate subgroups may lead to erroneous conclusions [56, 58], for example, the same treatment effect is observed in both biomarker subgroups but due to a difference in sample sizes, the effect is significant only in one of the subgroups.

Half of the reviewed studies analyzed more than one biomarker and/or endpoint, but only 3 studies corrected for multiple testing [40, 41, 46] in order to reduce the probability of false positive findings. This is important since the chance of false positive conclusions is already increased for underpowered studies [59].

Several guidelines provide suggestions for the analysis of treatment heterogeneity [10–14]. The most specific guideline for this subject is the PATH statement [60, 61], which recommends evaluating variation in the treatment effect by biomarker subgroup via interaction analysis. However, the PATH statement distinguishes between (i) risk modeling, i.e., combining all available prognostic factors into a prognostic score and evaluating interaction between this score and treatment and (ii) effect modeling, i.e., evaluating interactions between treatment and single predictive biomarkers [60]. None of the studies in this review performed risk modeling. Moreover, the PATH statement emphasizes that analyses of heterogeneity of relative effects can lead to different results than comparing absolute effects, i.e., risk differences, and the latter approach is recommended [60, 61]. All studies in this review, however, used relative effect measures, i.e., HRs or ORs, to investigate treatment heterogeneity on a multiplicative scale. Only one study presented, in addition to relative effects, an absolute measure of treatment benefit, namely the difference between the reduction in risk of recurrence [24]. Using relative effect measures is convenient because it is implemented in statistical software. However, absolute effect measures, i.e., risk differences, are considered to be more relevant for clinical decision making, and the evaluation of the treatment heterogeneity should therefore be based on absolute effects [60, 61].

Our review has several limitations. We restricted the detailed evaluation of studies to those published in 2019 in a subgroup of selected scientific journals. The initial selection of journals was based on our knowledge of relevant articles published in different years, with impact factors for 2020 ranging from 4.8 to 12.5. We added journals with higher and lower impact factors, whose scope included studies on predictive biomarkers for BC. While admittedly subjective, we believe that this sample of recent research in peer-reviewed established journals allows general conclusions about the entire field of BC research. A cursory review of publications in other journals and other years confirmed our results. We also assume that results on BC are to some extent generalizable to cancers at other sites.

Another potential limitation is that we may have missed studies with rarely used statistical methods. Most of the studies we reviewed used either biomarker-specific Kaplan-Meier plots of treatment effects and/or multiplicative interaction analyses. Only 3 studies used other methods (STEPP and a Bayesian covariate-adjusted logistic model). Alternative methods have been described in the literature but were not applied in the reviewed studies, e.g., the predictiveness curve for a continuous marker [62] or the metric theta which measures a difference in the disease rate under biomarker-based treatment assignment versus the default strategy of the same treatment for all patients [63]. Therefore, our review possibly does not capture the spectrum of less commonly used methods. However, finding all applied methods was not the goal of our work.

The strengths of our review include the comprehensive search which took into account all types of biomarkers and the thorough evaluation of eligible studies with regard to methodological features. Therefore, the importance of decent study design and adequate sample size is stressed.

The results of our review illustrate an important bottleneck in the development of new predictive tests. A new candidate test has to pass several stages of development and the most difficult step is the transition from a promising preclinical test to a test which can be applied to patients with cancer in daily practice. Series of patients are required to demonstrate the clinical utility of a test, including those who received the treatment of interest or an alternative treatment, and including patients with positive and negative test results. The large series required by current statistical methods are often not available, and if they are available, limited research budgets prohibit performing the test of interest. Consequently, too small patient series are interrogated leading to inconclusive results. It is likely that many promising test are erroneously abandoned at this stage of development.

Next to careful planning of biomarker-based studies of treatment heterogeneity, further research is necessary on statistical methods which allow evaluation of candidate predictive biomarkers with smaller numbers of patients than currently required for adequate statistical power. Case-only, hybrid designs or additive models may offer opportunities.

## Conclusions

This review shows that BC studies of predictive biomarkers are usually evaluated by separately estimating treatment effect in biomarker subgroup or by testing a multiplicative interaction term between biomarker and treatment with a regression analysis. These analyses may be underpowered because the studies are designed to investigate main treatment effects and biomarker data is often not available for all patients included in the study. Therefore, there is a need for further research on more powerful statistical methods which can be applied to small studies on predictive biomarkers.

### Electronic supplementary material

Below is the link to the electronic supplementary material.

Supplementary Material 1

Supplementary Material 2

## Data Availability

The data supporting the findings of this study are available within the reviewed articles that are publically available.

## Abbreviations

ACT: adjuvant chemotherapy
BC: breast cancer
ER: estrogen receptor
HER2: human epidermal growth factor 2
HoR: hormone receptor
HR: hazard ratio
OR: odds ratio
pCR: pathological complete response
PR: progesterone receptor
STEPP: subpopulation treatment effect pattern plot
TILs: tumor infiltrating lymphocytes
TNM: tumor, node, metastasis
