# Correction: A scoping review of statistical methods in studies of biomarker-related treatment heterogeneity for breast cancer

**DOI:** 10.1186/s12874-023-02030-3

**Published:** 2023-09-08

**Authors:** L Sollfrank, SC Linn, M Hauptmann, K Jóźwiak

**Affiliations:** 1 Institute of Biostatistics and Registry Research, Brandenburg Medical School Theodor Fontane, Fehrbelliner Straße 39, 16816 Neuruppin, Germany; 2 Division of Molecular Pathology, The Netherlands Cancer Institute, Amsterdam, The Netherlands; 3 Department of Medical Oncology, The Netherlands Cancer Institute, Amsterdam, The Netherlands; 4 Department of Pathology, University Medical Center, Utrecht, The Netherlands

**Correction: BMC Medical Research Methodology 23, 154 (2023)**

10.1186/s12874-023-01982-w

Following the publication of the original article [1], the authors noticed that the publisher had not updated the supplements after revision. They therefore requested to update the supplementary material 2.

The original article [1] has been updated.
