# Supplementary material for: A scoping review of statistical methods in studies of biomarker-related treatment heterogeneity for breast cancer
Source: BMC Med Res Methodol. 2023 Jun 29;23:154. doi: 10.1186/s12874-023-01982-w (PMC10308726; doi:10.1186/s12874-023-01982-w)
Supplement: Supplementary file 1 — Supplementary Material 1 [file 12874_2023_1982_MOESM1_ESM.docx]

**Additional file 1: Complete query**

The full query which we used for our PubMed search was the following:

(((((((((((("Annals of oncology : official journal of the European Society for Medical Oncology"[Journal]) OR ("Breast cancer research and treatment"[Journal])) OR ("Breast cancer research : BCR"[Journal])) OR ("International journal of cancer"[Journal])) OR ("Nature medicine"[Journal])) OR ("PloS one"[Journal])) OR ("Clinical cancer research : an official journal of the American Association for Cancer Research"[Journal])) OR ("Molecular cancer therapeutics"[Journal])) OR ("Journal of clinical oncology : official journal of the American Society of Clinical Oncology"[Journal])) OR ("JAMA"[Journal])) OR ("JAMA oncology"[Journal])) OR ("Journal of the National Cancer Institute"[Journal]) OR (("The Lancet. Oncology"[Journal]) OR ("Lancet (London, England)"[Journal]) OR ("The New England journal of medicine"[Journal]))) AND ("Breast Neoplasms"[Majr] OR ((breast[tiab] OR mammary[tiab]) AND (neoplas*[tiab] OR cancer*[tiab] OR tumor*[tiab] OR malignan*[tiab] OR oncolog*[tiab]))) AND (heterogeneity[TIAB] OR effect[TIAB] OR predict*[TIAB] OR prognostic[TIAB] OR interaction[TIAB]) AND (marker* OR biomarker*) AND (cohort[TIAB] OR patient*[TIAB] OR female[TIAB] OR women[TIAB]) AND (endocrine OR chemotherapy OR neoadjuvant)

In addition, a filter for 2019 as year of publication was applied.

We had knowledge of 7 papers published in different years which had met the eligibility criteria for our review [1-7]. It is reassuring that the query finds all of them.

References:

[1] Asleh K, Lyck Carstensen S, Tykjaer Jørgensen CL, Gao D, Won JR, Jensen M-B, et al. Basal biomarkers nestin and INPP4B predict gemcitabine benefit in metastatic breast cancer: Samples from the phase III SBG0102 clinical trial. Int J Cancer. 2019;144:2578–86.

[2] Vollebergh MA, Lips EH, Nederlof PM, Wessels LFA, Wesseling J, Vd Vijver MJ, et al. Genomic patterns resembling BRCA1- and BRCA2-mutated breast cancers predict benefit of intensified carboplatin-based chemotherapy. Breast Cancer Res. 2014;16:R47.

[3] Schouten PC, Marmé F, Aulmann S, Sinn H-P, van Essen HF, Ylstra B, et al. Breast Cancers with a *BRCA1* -like DNA Copy Number Profile Recur Less Often Than Expected after High-Dose Alkylating Chemotherapy. Clinical Cancer Research [Internet]. 2015;21:763–70.

[4] Schouten PC, Gluz O, Harbeck N, Mohrmann S, Diallo-Danebrock R, Pelz E, et al. BRCA1-like profile predicts benefit of tandem high dose epirubicin-cyclophospamide-thiotepa in high risk breast cancer patients randomized in the WSG-AM01 trial. Int J Cancer. 2016;139:882–9.

[5] van Rossum AGJ, Schouten PC, Weber KE, Nekljudova V, Denkert C, Solbach C, et al. BRCA1-like profile is not significantly associated with survival benefit of non-myeloablative intensified chemotherapy in the GAIN randomized controlled trial. Breast Cancer Res Treat. 2017;166:775–85.

[6] Severson TM, Wolf DM, Yau C, Peeters J, Wehkam D, Schouten PC, et al. The BRCA1ness signature is associated significantly with response to PARP inhibitor treatment versus control in the I-SPY 2 randomized neoadjuvant setting. Breast Cancer Res. 2017;19:99.

[7] Lips EH, Benard-Slagter A, Opdam M, Scheerman CE, Wesseling J, Hogervorst FBL, et al. BRCAness digitalMLPA profiling predicts benefit of intensified platinum-based chemotherapy in triple-negative and luminal-type breast cancer. Breast Cancer Res. 2020;22:79.
