# Supplementary material for: A scoping review of statistical methods in studies of biomarker-related treatment heterogeneity for breast cancer
Source: BMC Med Res Methodol. 2023 Jun 29;23:154. doi: 10.1186/s12874-023-01982-w (PMC10308726; doi:10.1186/s12874-023-01982-w)
Supplement: Supplementary file 2 — Supplementary Material 2 [file 12874_2023_1982_MOESM2_ESM.docx]

**Additional file 2: Supplementary tables**

**Supplementary Table** **1** Clinical characteristics of the studies included in the review

| Study | Number of patients | | Patients | Biomarker | Treatment | Endpoint | Type of study |
| --- | --- | --- | --- | --- | --- | --- | --- |
|  | Original study | Biomarker study |  |  |  |  |  |
| Adamo et al [22] | 61 | 54 | Postmenopausal, age ≥18, untreated, stage I–III HoR-pos/HER2-neg | Cont. PAM50-based CES | LTZ only vs mVNB (monotherapy/combination) | >50% relative decrease in 11-gene proliferation signature | Randomized |
| Alfarsi et al [46]^a^ | 1506  1592 | 377  271  294  329 | ER-pos | KIF18A mRNA expression (high vs low)  KIF18A protein expression (high vs low) | ET vs none | RFS  BCSS  RFS  BCSS | Observational |
| Asleh et al [23] | 337 | 239 | Locally advanced or metastatic | Basal defined as nestin+ or INPP4B-based on IHC analysis vs non-basal | GD vs D | OS | Randomized |
| Bartlett et al [24] | 6956 | 583 | HoR-pos, N pos, remained disease-free after ≥4 years of adjuvant TAM therapy | BCI [HOXB13 /IL17BR ratio (H/I)] (high vs low)  Cont. BCI | 10 vs 5 yrs of TAM | RFI | Randomized |
| Camp et al [25] | 1246 | 819 | N pos, operable | Cont. breast tumor dimension PC3 using PAM50 gene expression data  Cont. breast tumor dimension PC4 using PAM50 gene expression data | FEC-P vs FEC | DFS | Randomized |
| Chia et al [26] | 2840 | 617 | Age ≥18 (≥20 in Japan), stage I-IIIc (modified to stage II–IIIc in February 2010) operable, completion of (N)AC plus T ≤2 years before randomization, no evidence of disease recurrence or metastatic disease at study entry | PIK3CA altered (PCR PIK3CA mutated or FISH PIK3CA amplified or both) vs wild type | NET vs Pbo | iDFS | Randomized |
| Chien et al [52] | 151 | 151 | Age ≥18, stage II-III, no prior surgery or systemic treatment, tumor size ≥2.5 cm; HbA_1c_≤8; Not HoR-pos/HER2-neg with MammaPrint low risk | HoR (pos vs neg)  HER2 (pos vs neg) | MK-2206+Pac vs Pac | pCR | Randomized |
| Chumsri et al [45] | 3132  1415 | 2036  1415 | Primary, operable, N pos or high-risk N0 HER2-pos  Operable, N pos, HER2-pos | Age ≤40 vs >40 (at diagnosis) | ACTH vs ACT | RFS | Randomized |
| Chumsri et al [27] | 4547 | 3177 | High-risk HER2-pos | HoR (pos vs neg) | ACTH vs ACT | RFS | Randomized |
| Craze et al [47] | 1300 | 236 | TNM stage I-III, no T3-T4, sporadic primary operable invasive, age ≤70, TNBC | GLUD1 protein (high vs low) | CT vs no CT | BCSS | Observational |
| Dieci et al [28] | 1253 | 866 | HER2-pos | sTILs (≥20% vs <20%)  Cont. sTILs (in 10% increments) | Anthracycline+taxane-based CT with 9wks or 1yr of T | DDFS | Randomized |
| Gluz et al [21] | 336 | 306 | TN with cT1c-cT4c or clinically N-pos | Cont. FGFR4  Cont. PDL1 | Gem vs Carbo | pCR | Randomized |
| Hamy et al [29] | 340 | 155 | HER2-neg | PTGS2 (high vs low) | ECD+C vs ECD | EFS  OS | Randomized |
| Hrebien et al [48]^a^ | 148 | 42  59 | Advanced or metastatic ER-pos/HER2-neg, CT received | CDR28 (ctDNA) (high vs suppressed)  Clonal haematopoiesis of indeterminate potential (CHIP) (yes vs no) | C+Pac vs Pbo+Pac | PFS | Randomized |
| Ignatov et al [49] | 442 | 251 | Invasive non-metastatic | GPER-1 (pos vs neg) | TAM vs AI | DFS | Observational |
| Janning et al [30] | 1948 | 1160 | Untreated, histologically confirmed HER2-neg, invasive, non-metastatic, cT3 or cT4, TNBC or ER-pos/PR-pos, palpable nodes (cN+ for cT2) or pos findings on sentinel-node-biopsy (pNSLN pos for cT1) | sCAIX (cont.)  sCAIX (high vs low)  sCAIX (cont.)  sCAIX (high vs low)  sCAIX (cont.) | NCT vs NCT-B | pCR  DFS  OS | Randomized |
| Kensler et al [31] | 8010 | 1753 | Postmenopausal, early stage HoR-pos | AR (pos vs neg)  Cont. AR | LTZ vs TAM monotherapy | BCFI  DFS  BCFI | Randomized |
| Kruger et al [32]^a^ | 1662 | 367  432 | Postmenopausal, stage I-III, ER-pos | p-IGF-1R (pos vs neg)  p-ERα (Ser118) (pos vs neg) | TAM vs CONV | RFI | Randomized |
| Kubo et al [42] | 238711 | 2735 | pT1N0M0, HER2-pos, iDC | pT1a vs pT1b vs pT1c | Treated (CT with or without T) vs untreated | DFS  OS | Observational |
| Loibl et al [33]^a^ | 174 | 174  158 | Previously untreated uni- or bilateral primary, non-metastatic invasive TNBC, cT2-cT4a-d, TNBC, determination of sTILs, PD-L1, Ki-67 | sTIL (low vs intermediate vs high)  PD-L1 (pos vs neg) | Nab-P+EC+Dur vs Nab-P+EC+Pbo | pCR | Randomized |
| Loibl et al [34] | 1206 | 851  295 | cT2-cT4a-d or cT1a and at least one additional risk factor: clinical or pathological (pos sentinel node biopsy in case of cN0 or core biopsy in cN pos) nodal involvement, HoR-neg, HER2-pos, or Ki-67≥20%  HER2-pos subgroup | PIK3CA (mutated vs wild type) | Nab-P vs Pac | pCR | Randomized |
| Mori et al [43] | 242 | 241 | TNBC | T-bet (pos vs neg) | ACT vs none | RFS  OS | Observational |
| Nanni et al [35] | 126 | 122 | Metastatic HER2, stage IV, non-endocrine-responsive disease, measurable/non-measurable disease according to RECIST Criteria, availability of HOMA index, age 18-75, ECOG PS ≥2, normal organ and bone marrow functions, LVEF >50% | HOMA index ≥2.5 vs <2.5 | M+CT vs CT | OS  PFS | Randomized |
| Nitz et al [36] | 2449 | 1791 | Age 18-75, HER2-neg, pT1-pT4c, pN pos or pN0 (if ≥pT2, grade 2/3, high urokinase-type plasminogen activator/plasminogen activator inhibitor, <35 age, or HoR-neg), Eastern Cooperative Oncology Group performance status <2 or Karnofsky status ≥80%; HoR-pos EBC with ≥4 lymph nodes or RS >11 | RS >25 vs ≤25 | ETC vs TC | DFS | Randomized |
| Pizzutti et al [44] | 738 | 707 | HER2-pos, metastatic | Triple-pos vs ER-pos or PR-pos vs HoRs-neg | Pert vs no Pert | PFS | Observational |
| Puppe et al [37] | 885 | 161 | Tumor-pos lymph nodes, HER2-neg | BRCA1-like, or non-BRCA1-like & EZH2≤50% vs non-BRCA1-like & EZH2>50% | HD CT vs CONV | DFS  OS | Randomized |
| Sestak et al [38] | 15146 | 3746 | ER-pos, HER2-neg | Cont. Epclin | ET vs ET+CT | DRFI  BCFI | Observational |
| Siddappa et al [50] | 70 | 70 | Test cohort: stage II/III; validation cohort: stage II/III TNBC | ERBB2 (pos vs neg) | T vs no T | MRFS | Observational |
| Swain et al [39] | 529 | 176 | HER2-pos | HER2E vs Basal-like/LumA/LumB | T+L vs T | pCR | Randomized |
| Szijgyarto et al [40]^a^ | 4724 | 673  624 | Postmenopausal | pT202/T204MAPK (≥10% vs 0%)  pS167ERα (≥10% vs 0%) | TAM vs EX | DFS  OS  DFS  OS | Randomized |
| Turner et al [41]^a^ | 521 | 302  226 | HoR-pos, metastatic | CCNE1 (high vs low)  LumA vs LumB | Pal+Ful vs Pbo+Ful | PFS | Randomized |

**Abbreviations:** ACT: adjuvant CT; ACTH: ACT with T; AI: aromatase inhibitor; B: bevacizumab; BCFI: invasive breast cancer-free interval; BCFS: breast cancer-free survival; BCI: breast cancer index; BCSS: breast cancer specific survival; C: capivasertib; Carbo: carboplatin; cont.: continuous; CES: chemo-endocrine score; CONV: conventional CT; CT: chemotherapy; D: doctaxel; DFS: disease-free survival; DDFS: distant disease-free survival; DRFI: distant recurrence-free interval; Dur: durvalumab; EBC: early breast cancer; EC: epirubicin+cycloposhamide; ECD: EC+D; ECD+C: ECD with celecoxib; EFS: event-free survival; ER: estrogen receptor; ET: endocrine treatment; ETC: 4 cycles of epirubicin (E)_90_/cyclophosphamide (C)_600_ followed by 4 cycles of docetaxel (T)_100_; EX: Exemstane; FEC: 6 cycles of adjuvant fluorouracil, epirubicin, and cyclophosphamide; FEC-P: FEC followed by 8 weekly cycles of Pac; Ful: fulvestrant; GD: gemcitabine-docetaxel; Gem: gemcitabine; HD: high-dose; HER2: human epidermal growth factor receptor 2; HER2E: HER2 enriched; HoR: hormone receptor; iDFS: invasive disease-free survival; L: lapatinib; LTZ: letrozole; Lum: luminal; M: metastatic; +M: metformin; MRFS: metastatic recurrence-free survival; N: nodal status; NAB-P: nab-Pac; NCT: neoadjuvant CT; neg: negative; NET: neratinib; OS: overall survival; Pac: paclitaxel; Pal: Palbociclib; Pbo: Placebo; pCR: pathological complete response; Pert: pertuzumab; PFS: progression-free survival; pos: positive; PR: progesterone receptor; pT/cT: tumor stage; RFI: recurrence-free interval; RFS: recurrence-free survival; RS: recurrence score; sTILs: stromal tumor infiltrating lymphocytes; T: trastuzumab; TAM: Tamoxifen; TC: 6 cycles ofT_75_C_600_; TN: triple negative; TNBC: triple negative breast cancer; TNM: tumor, node, metastasis; vs: versus; wks: weeks, yr: year

^a^ Number of patients in different analyses varied due to incomplete patient information, e.g. lost to follow-up, missing information on biomarker measurements

***Supplementary Table 2*** *Statistical characteristics of the studies included in the review*

| Study | Subgroup: # patients (# events)^a^ | Median Follow up time^b^ | Endpoint | Type of interaction analysis^c^ | Type of interaction | Type of model^d^ |
| --- | --- | --- | --- | --- | --- | --- |
| Adamo et al [22] | LTZ only: 20 (11) mVNB arms: 34 (18) | N. a. | >50% relative decrease in 11-gene proliferation signature | Logistic regression with cont. biomarker*treatment interaction | Multiplicative, qualitative:  p=0.039 | B |
| Alfarsi et al [46] | KIF18A mRNA high/ET: 193 (-) KIF18A mRNA high/None: 59 (-) KIF18A mRNA low/ET: 78 (-) KIF18A mRNA low/None: 47 (-)  KIF18A mRNA high/ET: 153 (-) KIF18A mRNA high/None: 31 (-) KIF18A mRNA low/ET: 58 (-) KIF18A mRNA low/None: 29 (-)  KIF18A high/ET: 75 (-)  KIF18A high/None: 87 (-)  KIF18A low/ET: 62 (-)  KIF18A low/None: 70 (-)  KIF18A high/ET: 80 (-) KIF18A high/None: 99 (-) KIF18A low/ET: 65 (-) KIF18A low/None: 85 (-) | N. r. | RFS  OS  RFS  OS | KM plots for biomarker effect by treatment subgroups  KM plots for biomarker effect by treatment subgroups  KM plots for biomarker effect by treatment subgroups  KM plots for biomarker effect by treatment subgroups | No test of interaction  No test of interaction  No test of interaction  No test of interaction | C-2 |
| Asleh et al [23] | Basal/GD: 21 (-)  Basal/D: 20 (-)  Non-basal/GD: 102 (-)  Non-basal/D: 96 (-) | 13 yrs | OS | Cox model with binary biomarker*treatment interaction; subgroup analysis with KM for treatment effect in biomarker subgroups: HR in basal group: 0.31 (0.16-0.60), HR in non-basal group: 0.99 (0.73-1.34) | Multiplicative, qualitative:  p<0.010 | A, C-1 |
| Bartlett et al [24] | BCI high/10yrs: 150 (-)  BCI high/5yrs: 137 (-)  BCI low/10yrs: 141 (-)  BCI low/5yrs: 155 (-)  10 yrs TAM: 291 (77)  5 yrs TAM: 292 (92) | 8.9 yrs | RFI | KM plots and subgroup analysis for treatment effect in biomarker subgroups: HR in BCI high: 0.35 (0.15-0.86), HR in BCI low: 1.07 (0.69-1.65); absolute treatment benefit in BCI high: 10.2%, absolute treatment benefit in BCI low: -0.2%  Cox model with cont. biomarker*treatment interaction | No test of interaction  Multiplicative, qualitative:  p=0.024 | A, C-1, E |
| Camp et al [25] | FEC-P: 402 (-)  FEC: 417 (-) 283 events in total | 8.7 yrs | DFS | Cox model with cont. biomarker*treatment interaction: treatment HR: 0.75 (0.59-0.95); interaction HR: 1.26 (1.00-1.60)  Cox model with cont. biomarker*treatment interaction: treatment HR: 0.73 (0.58-0.93); interaction HR: 1.28 (1.00-1.62) | Multiplicative:  p=0.052  Multiplicative, qualitative:  p=0.048 | A |
| Chia et al [26] | Altered/NET: 130 (8) Altered/Pbo: 132 (20) Wild/NET: 175 (14) Wild/Pbo: 180 (20) | N. r. | iDFS | Cox model with binary biomarker*treatment interaction; subgroup analysis for treatment effect by biomarker subgroup: HR in PIK3CA-altered: 0.41 (0.17–0.90), HR in PIK3CA- wild type: 0.72 (0.36–1.41) | Multiplicative:  p=0.309 | A, C-1 |
| Chien et al [52] | HoR-pos/MK-2206: 44 (-)  HoR-pos/Pac: 30 (-)  HoR-neg/MK-2206: 50 (-)  HoR-neg/Pac: 27 (-)  HER2-pos/MK-2206: 34 (-)  HER2-pos/Pac: 60 (-)  HER2-neg/MK-2206: 10 (-)  HER2-neg/Pac: 47 (-) | N. a. | pCR | Bayesian covariate-adjusted logistic model for calculation of probability of superiority of experimental treatment over standard treatment: for HoR-pos: 81.9%; for HoR-neg: 98.5%  Bayesian covariate-adjusted logistic model for calculation of probability of superiority of experimental treatment over standard treatment: for HER2-pos: 94.3%; HER2-neg: 95.2% | No test of interaction  No test of interaction | D |
| Chumsri et al [45] | Young/ACT: 226 (63) Young/ACTH: 170 (32) Old/ACT: 861 (193) Old/ACTH: 779 (140)  Young/ACT: 149 (64)  Young/ACTH: 131 (33)  Old/ACT: 557 (174)  Old/ACTH: 578 (105) | 10.3 yrs  8 yrs | RFS  RFS | Cox model with binary biomarker*treatment interaction; subgroup analysis and KM plots for treatment effect by biomarker groups: HR in young: 0.43 (0.28-0.66), HR in old: 0.56 (0.45-0.69)  Cox model with binary biomarker*treatment interaction; subgroup analysis and KM plots for treatment effect by biomarker groups: HR in young: 0.45 (0.29-0.68), HR in old: 0.42 (0.33-0.54) | Multiplicative:  p=0.270  Multiplicative:  p=0.820 | A, C-1 |
| Chumsri et al [27] | HoR-pos/ACTH: 826 (141)  HoR-pos/ACT: 905 (240)  HoR-neg/ACTH: 700 (145)  HoR-neg/ACT: 746 (238) | 8.0 yrs in non-cases | RFS | KM plots, hazard rates, x-years RFS by treatment and biomarker and subgroup analysis for treatment effect in biomarker subgroup: HR in HoR-pos: 0.46 (0.37-0.57), HR in HoR-neg: 0.47 (0.38-0.58) | Multiplicative:  p=0.870 | C-1 |
| Craze et al [47] | High: 32 (-)  Low: 197 (-) | N. r. | BCSS | KM plots and log-rank test within treatment subgroups | No test of interaction | C-2 |
| Dieci et al [28] | TILs high/9wks: 98 (2)  TILs high/1yr: 96 (8)  TILs low/9wks: 327 (45)  TILs low/1yr: 345 (28)  9wks: 425 (47)  1 yr: 441 (36) | 6.1 yrs | DDFS | Cox model with binary biomarker*treatment interaction; KM plots, log-rank test and subgroups analysis for treatment effects in biomarker subgroups: HR for TILs low: 1.75 (1.09-2.80); HR for TILs high: 0.23 (0.05-1.09)  Cox model with cont. biomarker*treatment interaction: HR in 9wks: 0.60 (0.41-0.88), HR in 1yr: 0.89 (0.71-1.12) | Multiplicative, qualitative:  p=0.015  Multiplicative:  p=0.085 | A, C-2 |
| Gluz et al [21] | Gem: 169 (-) Carbo: 137 (-) in total 109 events | N. a. | pCR | Logistic model with cont. biomarker*treatment interaction: marker OR: 0.22 (0.11-0.47), interaction OR: 2.84 (1.72-4.70)  Logistic model with cont. biomarker*treatment interaction: marker OR: 1.97 (1.01-3.82), interaction OR: 2.60 (1.63-4.14) | Multiplicative, quantitative:  p<0.001  Multiplicative, quantitative:  p<0.001 | B |
| Hamy et al [29] | PTGS2 high/ECD+C: 24 (8) PTGS2 high/ECD: 28 (13) PTGS2 low/ECD+C: 54 (27) PTGS2 low/ECD: 49 (10)  PTGS2 high/ECD+C: 24 (6) PTGS2 high/ECD: 28 (9) PTGS2 low/ECD+C: 54 (17) PTGS2 low/ECD: 49 (5) | N. r. | EFS  OS | Cox model with binary biomarker*treatment interaction; KM plots, log-rank test and subgroup analysis for treatment effect by biomarker subgroups: HR in PTGS2 high: 0.75 (0.30-1.83), HR in PTGS2 low: 3.01 (1.45-6.24)  Cox model with binary biomarker*treatment interaction; KM plots, log-rank test and subgroup analysis for treatment effect by biomarker subgroups: HR in PTGS2 high: 0.80 (0.28-2.24), HR in PTGS2 low: 3.32 (1.23-9.01) | Multiplicative, qualitative:  p=0.010  Multiplicative: p n.r. | A, C-1 |
| Hrebien et al [48] | High/C: 5 (4) High/Pbo: 5 (5) Sup/C: 16 (11) Sup/Pbo: 16 (13)  Yes/C: 9 (8)  Yes/Pbo: 9 (6)  No/C: 19 (17)  No/Pbo: 22 (17) | N. r. | PFS | KM plots, log-rank test and subgroup analysis for biomarker effect in treatment subgroups; HR in C: 0.11 (0.03-0.41), HR in Pbo: 0.37 (0.11-1.28)  KM plots, log-rank test and subgroup analysis for biomarker effect in treatment subgroups; HR in C: 0.80 (0.33-1.93), HR in Pbo: 1.35 (0.52-3.50) | No test of interaction  No test of interaction | C-2 |
| Ignatov et al [49] | TAM: 137 (-) AI: 114 (-) 40 events in total | 33 months | DFS | KM plots and log-rank test for biomarker effect in treatment subgroups | No test of interaction | C-2 |
| Janning et al [30] | NCT-B: 577 (108)  NCT: 583 (102)  sCAIX high/NCT-B: 353 (62)  sCAIX high/NCT: 360 (75)  sCAIX low/NCT-B: 224 (46)  sCAIX low/NCT: 223 (27)  NCT-B: 577 (-)  NCT: 583 (-)  sCAIX high/NCT-B: 353 (-)  sCAIX high/NCT: 360 (-)  sCAIX low/NCT-B: 224 (-)  sCAIX low/NCT: 223 (-)  NCT-B: 577 (-)  NCT: 583 (-) | N a.  N. r. | pCR  DFS  OS | Logistic regression with cont. biomarker *treatment interaction; subgroup analysis for biomarker effect by treatment subgroup: OR in NCT-B: 0.92 (0.71-1.23), OR in NCT: 1.58 (1.21-2.12)  Logistic regression with binary biomarker*treatment interaction; subgroup analysis for treatment effect by biomarker subgroup: OR in sCAIX high: 0.80 (0.58-1.36), OR in sCAIX low: 2.16 (1.24-3.88)  Cox model with cont. biomarker*treatment interaction; subgroup analysis of biomarker effect by treatment subgroup: HR in NCT-B: 1.15 (0.90-1.50), HR in NCT: 0.81 (0.67-0.99)  KM plots and subgroup analysis for treatment effect in biomarker subgroups: HR in sCAIX high: 1.47 (1.04-2.08), HR in sCAIX low: 0.80 (0.52-1.22)  Cox model with cont. biomarker*treatment interaction; subgroup analysis of biomarker effect by treatment subgroup: HR in NCT-B: 1.07 (0.80-1.47), HR in NCT: 0.86 (0.64-1.15) | Multiplicative, qualitative: p=0.007  Multiplicative, qualitative: p=0.012  Multiplicative, qualitative: p=0.033  No test of interaction  Multiplicative: p=0.293 | A, B, C-1, C-2 |
| Kensler et al [31] | AR-pos/LTZ: 738 (-)  AR-pos/TAM: 690 (-)  AR-neg/LTZ: 148 (-)  AR-neg/TAM: 177 (-)  236 (BCFI) and 401 (DFS events in total) | 8 yrs | BCFI  DFS  BCFI | Cox model with binary biomarker*treatment interaction; KM plots and subgroup analysis for treatment effect by biomarker subgroups: HR in AR-pos: 0.73 (0.55-0.98), HR in AR-neg: 0.49 (0.27-0.87)  Cox model with binary biomarker*treatment interaction; subgroup analysis for treatment effect by biomarker subgroups: HR in AR-pos: 0.80 (0.64-0.99), HR in AR-neg: 0.61 (0.39-0.94)  STEPP | Multiplicative: p=0.220  Multiplicative: p=0.270  No test of interaction | A, C-1, STEPP |
| Kruger et al [32] | p-IGF-1R-pos/TAM: 97 (-)  p-IGF-1R-pos/none: 25 (-)  p-IGF-1R-neg/TAM: 185 (-)  p-IGF-1R-neg/none: 57 (-)  90 events in total  p-ERα (Ser118)-pos/TAM: 135 (-)  p-ERα (Ser118)-pos /none: 40 (-)  p-ERα (Ser118)-neg TAM: 205 (-)  p-ERα (Ser118)-neg /none: 52 (-)  109 events in total | 8.4 yrs | RFS | Cox model with binary biomarker*treatment interaction; KM plots and subgroup analysis for treatment effect by biomarker subgroups: HR in p-IGF-1R-pos: 0.95 (0.27-3.38), HR in p-IGF-1R-neg: 0.41 (0.22-0.75)  Cox model with binary biomarker*treatment interaction; KM plots and subgroup analysis for treatment effect by biomarker subgroup: HR in p-ERα (Ser118)-neg: 0.50 (0.27–0.93), HR in p-ERα (Ser118)-pos: 0.72 (0.29–1.80) | Multiplicative: p=0.230  Multiplicative: p=0.510 | A, C-1 |
| Kubo et al [42] | pT1a/treated: 120 (-)  pT1a/untreated: 369 (-)  pT1b/treated: 324 (-)  pT1b/untreated: 300 (-)  pT1c/treated: 1028 (-)  pT1c/untreated: 594 (-)  212 (DFS) and 80 (OS) events in total | 76 months | DFS  OS | KM plots, log-rank test and subgroup analysis for treatment effects in biomarker subgroup: HR in pT1c: 0.47 (0.32-0.68), HR in pT1b and in pT1a: n. r.  KM plots and subgroup analysis for treatment effects in biomarker subgroup: HR in pT1c: 0.54 (0.31-0.94), HR in pT1b: 0.20 (0.06-0.67), HR in pT1a: n. r. | No test of interaction  No test of interaction | C-1 |
| Loibl et al [33] | sTIL high/Dur: 12 (-) sTIL high/Pbo: 13 (-) sTIL inter/Dur: 42 (-) sTIL inter/Pbo: 41 (-) sTIL low/Dur: 34 (-) sTIL low/Pbo: 32 (-)  PD-L1-pos/Dur: 69 (40) PD-L1-pos/Pbo: 69 (35) PD-L1-neg/Dur: 9 (4) PD-L1-neg/Pbo: 11 (2) | N. a. | pCR | Logistic regression with categorical biomarker*treatment interaction; subgroup analysis for treatment effect in biomarker subgroup: OR in sTIL low: 1.54 (0.56-4.24), OR in sTIL inter: 1.40 (0.59-3.32), OR in sTIL high: 2.22 (0.33-15.2)  Logistic regression with binary biomarker*treatment interaction; subgroup analysis for treatment effect in biomarker subgroup: OR in PD-L1-pos: 1.34 (0.68, 2.62), OR in PD-L1-neg: 3.60 (0.48-27.10) | Multiplicative: p=0.912  Multiplicative: p=0.363 | B, C-1 |
| Loibl et al [34] | PIK3CA-mut/NAB-P: 90 (19) PIK3CA-mut/Pac: 93 (23) PIK3CA-wild/NAB-P: 347 (151) PIK3CA-wild/Pac: 321 (108)  PIK3CA-mut/NAB-P: 31 (12) PIK3CA-mut/Pac: 32 (18) PIK3CA-wild/NAB-P: 120 (86) PIK3CA-wild/Pac: 112 (68) | N. a. | pCR | Logistic regression with binary biomarker*treatment interaction; subgroup analysis for treatment effect in biomarker subgroup: OR in PIK3CA-mut: 0.81 (0.41-1.63), OR in PIK3CA-wild type: 1.52 (1.11-2.08)  Logistic regression with binary biomarker*treatment interaction; subgroup analysis for treatment effect in biomarker subgroup: OR in PIK3CA-mut: 0.49 (0.18-1.34), OR in PIK3CA-wild type: 1.64 (0.95-2.83) | Multiplicative: p>0.050  Multiplicative, qualitative: p=0.007 | B, C-1 |
| Mori et al [43] | T-bet-pos/ACT: 50 (-)  T-bet-pos/none: 17 (-)  T-bet-neg/ACT: 117 (-)  T-bet-neg/none: 57 (-) | 67 months | RFS | KM plots, log-rank test and subgroup analysis for treatment effect by biomarker subgroups | No test of interaction | C-1 |
| Nanni et al [35] | HOMA ≥2.5/CT+M: 29 (-)  HOMA ≥2.5/CT: 28 (-)  HOMA <2.5/CT+M: 28 (-)  HOMA <2.5/CT: 37 (-)  71 events in total  HOMA ≥2.5/CT+M: 29 (-)  HOMA ≥2.5/CT: 28 (-)  HOMA <2.5/CT+M: 28 (-)  HOMA <2.5/CT: 37 (-)  112 events in total | 39.6 months | OS  PFS | Cox model with binary biomarker*treatment interaction  Cox model with binary biomarker*treatment interaction; subgroup analysis for treatment effect by biomarker subgroups: HR in HOMA ≥2.5: 1.01 (0.57-1.76), HR in HOMA <2.5: 1.03 (0.61-1.73) | Multiplicative: p=0.942  Multiplicative: p=0.997 | A, C-1 |
| Nitz et al [36] | RS >25/TC: 254 (36)  RS >25/EC-T: 231 (37)  RS ≤25/TC: 657 (37)  RS ≤25/EC-T: 649 (33) | 60 months | DFS | Cox model with binary biomarker*treatment interaction; KM plots and subgroup analysis for treatment effect by biomarker subgroups: HR in RS ≤25: 1.13, HR in RS >25: 0.87 | Multiplicative:  p>0.050 | A, C-1 |
| Pizzutti et al [44] | Pert: 371 (-) no-Pert: 336 (-) biomarker proportions not reported | 32.9 months | PFS | KM plots and log-rank test for treatment effect within biomarker subgroups | No test of interaction | C-1 |
| Puppe et al [37] | BRCA1/HD: 18 (6) BRCA1/CONV: 19 (16) Non-BRCA1&EZH2high/HD: 40 (15) Non-BRCA1&EZH2high/CONV: 31 (20)  Non-BRCA1&EZH2low/HD: 28 (15) Non-BRCA1&EZH2low/CONV: 25 (12)  BRCA1/HD: 18 (4) BRCA1/CONV: 19 (13) Non-BRCA1&EZH2high/HD: 40 (8) Non-BRCA1&EZH2high/CONV: 31 (17)  Non-BRCA1&EZH2low/HD: 28 (9) Non-BRCA1&EZH2low/CONV: 25 (9) | N. r. | DFS  OS | Cox model with categorical biomarker*treatment interaction; KM plots, log-rank test and subgroup analysis for treatment effect by biomarker subgroup: HR in BRCA1: 0.18 (0.07-0.48), HR in non-BRCA1/EZH2high: 0.35 (0.17-0.71), HR in non-BRCA1/EZH2low: 0.94 (0.44-2.03)  Cox model with categorical biomarker*treatment interaction; KM plots, log-rank test and subgroup analysis for treatment effect by biomarker subgroup: HR in BRCA1: 0.21 (0.06-0.65), HR in non-BRCA1/EZH2high: 0.31 (0.13-0.72), HR in non-BRCA1/EZH2low: 0.71 (0.28-1.80) | Multiplicative, qualitative:  p=0.015  Multiplicative:  p=0.115 | A-C-1 |
| Sestak et al [38] | ET: 2630 (279) ET+CT: 1116 (146)  ET: 2630 (398) ET+CT: 1116 (171) | N. r. | DRFI  BCFI | Cox model with cont. biomarker*treatment interaction; subgroup analysis for biomarker effect by treatment group: HR in ET: 2.79 (2.49-3.13), HR in ET+CT: 2.27 (1.99-2.59)  Cox model with cont. biomarker*treatment interaction, subgroup analysis for biomarker effect by treatment group: HR in ET: 2.50 (2.26-2.76), HR in ET+CT: 2.06 (1.82-2.34) | Multiplicative, quantitative: p=0.022  Multiplicative, quantitative:  p=0.025 | A, C-2 |
| Siddappa et al [50] | ERBB2-pos/T: 4 (1) ERBB2-pos/NoT: 4 (3) ERBB2-neg/T: 15 (4) ERBB2-neg/NoT: 47 (14) | N. r. | MRFS | KM plots for subgroups | No test of interaction | C-3 |
| Swain et al [39] | HER2E/T+L: 57 (40)  HER2E/T: 71 (46)  other/T+L: 25 (6)  other/T: 23 (4) | N.a. | pCR | Logistic model with binary biomarker*treatment interaction; subgroup analysis for treatment effect in biomarker subgroups: OR in HER2E: 1.29 (0.60-2.77), OR in other: 1.37 (0.33-5.74) | Multiplicative:  p=0.940 | B, C-1 |
| Szijgyarto et al [40] | pT202/T204MAPK ≥10%/EX: 153 (39)  pT202/T204MAPK ≥10%/TAM: 161 (44)  pT202/T204MAPK 0%/EX: 199 (55)  pT202/T204MAPK 0%/TAM: 160 (54)  pT202/T204MAPK ≥10%/EX: 153 (25)  pT202/T204MAPK ≥10%/TAM: 161 (28)  pT202/T204MAPK 0%/EX: 199 (43)  pT202/T204MAPK 0%/TAM: 160 (41)  pS167ERα ≥10%/EX: 164 (37)  pS167ERα ≥10%/TAM: 165 (47)  pS167ERα 0%/EX: 162 (39)  pS167ERα 0%/TAM: 133 (42)  pS167ERα ≥10%/EX: 164 (24)  pS167ERα ≥10%/TAM: 165 (27)  pS167ERα 0%/EX: 162 (31)  pS167ERα 0%/TAM: 133 (36) | 91 months | DFS  OS  DFS  OS | Cox model with binary biomarker*treatment interaction; KM plots, log-rank test and subgroup analysis for treatment effect by biomarker subgroup: HR in pT202/T204MAPK ≥10%: 0.92 (0.60-1.41), HR in pT202/T204MAPK 0%: 0.77 (0.53-1.12)  Cox model with binary biomarker*treatment interaction; KM plots, log-rank test and subgroup analysis for treatment effect by biomarker subgroup: HR in pT202/T204MAPK ≥10%: 0.96 (0.56-1.65), HR in pT202/T204MAPK 0%: 0.83 (0.54-1.27)  Cox model with binary biomarker*treatment interaction; KM plots, log-rank test and subgroup analysis for treatment effect by biomarker subgroup: HR in pS167ERα ≥10%: 0.79 (0.51-1.22), HR in pS167ERα 0%: 0.70 (0.45-1.08)  Cox model with binary biomarker*treatment interaction; KM plots, log-rank test and subgroup analysis for treatment effect by biomarker subgroup: HR in pS167ERα ≥10%: 0.93 (0.53-1.61), HR in pS167ERα 0%: 0.66 (0.41-1.06) | Multiplicative:  p=0.810  Multiplicative:  p=0.750  Multiplicative:  p=0.810  Multiplicative:  p=0.750 | A, C-1 |
| Turner et al [41] | CCNE1 high/Pal: 91 (-) CCNE1 high/Pbo: 60 (-) CCNE1 low/Pal: 103 (-) CCNE1 low/Pbo: 48 (-)  LumA/Pal: 83 (-) LumA/Pbo: 50 (-) LumB/Pal: 61 (-) LumB/Pbo: 32 (-) | N. r. | PFS | Cox model with binary biomarker*treatment interaction; STEPP, KM plots and subgroup analysis for treatment effect in biomarker subgroup: HR in CCNE1 high: 0.85 (0.58-1.26), HR in CCNE1 low: 0.32 (0.20-0.50)  Cox model with binary biomarker*treatment interaction; KM plots within biomarker subgroups; HR in LumA: 0.41 (0.25-0.66), HR in LumB: 0.64 (0.38-1.09) | Multiplicative, qualitative:  p=0.024  Multiplicative:  p=0.200 | A, C-1, STEPP |

**Abbreviations**: ACT: adjuvant CT; ACTH: ACT with T; AI: aromatase inhibitor; B: bevacizumab; BCFI: invasive breast cancer-free interval; BCFS: breast cancer-free survival; BCI: breast cancer index; BCSS: breast cancer specific survival; C: capivasertib; Carbo: carboplatin; cont.: continuous; CONV: conventional CT; CT: chemotherapy; D: doctaxel; DDFS: distant disease-free survival, DFS: disease-free survival; DRFI: distant recurrence-free interval; Dur: durvalumab; EBC: early breast cancer; EC: epirubicin+cycloposhamide; ECD: EC+D; ECD+C: ECD with celecoxib; ET: endocrine treatment; ETC: 4 cycles of epirubicin (E)_90_/cyclophosphamide (C)_600_ followed by 4 cycles of docetaxel (T)_100_; EFS: event-free survival; ER: estrogen receptor; EX: Exemstane; FEC: 6 cycles of adjuvant fluorouracil, epirubicin, and cyclophosphamide; FEC-P: FEC followed by 8 weekly cycles of Pac; Ful: fulvestrant; Gem: gemcitabine; GD: gemcitabine-docetaxel; HD: high-dose; HER2: human epidermal growth factor receptor 2; HER2E: HER2 enriched; HoR: hormone receptor; HR: Hazard ratio; iDFS: invasive disease-free survival; inter: intermediate; KM: Kaplan-Meier; L: lapatinib; LTZ: letrozole; Lum: luminal; M: metastatic; +M: metformin; mut: mutated; MRFS: metastatic recurrence-free survival; N: nodal status; n. a.: not applicable; NAB-P: nab-Pac; n. c.: not calculated; NCT: neoadjuvant CT; neg: negative; NET: neratinib; n. r.: not reported; OR: odds ratio; OS: overall survival; Pal: Palbociclib; Pac: paclitaxel; Pbo: Placebo; pCR: pathological complete response; Pert: pertuzumab; PFS: progression-free survival; pos: positive; PR: progesterone receptor; pT/cT: tumor stage; RFI: recurrence-free interval; RFS: recurrence-free survival; RS: recurrence score; STEPP: Subpopulation treatment effect pattern plot; sTILs: stromal tumor infiltrating lymphocytes; T: trastuzumab; TAM: Tamoxifen; TC: 6 cycles ofT_75_C_600_; wild: wild type; wks: weeks, yr: year

^a^ Given in the form ‘biomarker/treatment: number of patients (number of events)’, first treatment proportions in biomarker level high group, then in biomarker level low; (-): number of events in particular subgroup was not reported

^b^ Not applicable for binary endpoints

^c^ 95% confidence interval for each HR/OR in parentheses

^d^ A: Cox model with multiplicative biomarker-treatment interaction, B: logistic model with multiplicative biomarker-treatment interaction, C-1: subgroup analysis for treatment effect by biomarker subgroups, C-2: subgroup analysis for biomarker effect by treatment subgroups, C-3: subgroup analysis for all biomarker-treatment subgroups, STEPP: subpopulation treatment effect pattern plot, D: Bayesian covariate-adjusted logistic model, E: absolute treatment benefit by difference in cumulative incidence at one time point

***Supplementary Table 3*** *Multiplicity of hypotheses testing in breast cancer studies of predictive biomarkers*

| Author | Number of biomarkers^a^ | Number of endpoints^b^ | Number of (sub)populations^c^ | Total number of conducted analyses^d^ | Number of significant results |
| --- | --- | --- | --- | --- | --- |
| Adamo et al [22] | 2 | 1 | 1 | 2 | 1 |
| Alfarsi et al [46] | 2 | 3 | 1 | 6 | 6 |
| Asleh et al [23] | 1 | 1 | 1 | 1 | 1 |
| Bartlett et al [24] | 4 | 1 | 1 | 4 | 4 |
| Camp et al [25] | 5 | 1 | 1 | 5 | 1 |
| Chia et al [26] | 1 | 1 | 1 | 1 | 0 |
| Chien et al [52] | 3 | 1 | 1 | 3 | 2 |
| Chumsri et al [45] | 1 | 1 | 6 | 6 | 0 |
| Chumsri et al [27] | 1 | 1 | 1 | 1 | 0 |
| Craze et al [47] | 1 | 1 | 1 | 1 | 1 |
| Dieci et al [28] | 2 | 1 | 1 | 2 | 1 |
| Gluz et al [21] | 18 | 1 | 1 | 20 | 2 |
| Hamy et al [29] | 1 | 2 | 3 | 6 | 4 |
| Hrebien et al [48] | 2 | 1 | 1 | 2 | 1 |
| Ignatov et al [49] | 1 | 1 | 1 | 1 | 1 |
| Janning et al [30] | 2 | 3 | 3 | 15 | 7 |
| Kensler et al [31] | 2 | 2 | 1 | 3 | 0 |
| Kruger et al [32] | 3 | 1 | 1 | 3 | 0 |
| Kubo et al [42] | 1 | 3 | 1 | 3 | 3 |
| Loibl et al [33] | 6 | 1 | 1 | 6 | 1 |
| Loibl et al [34] | 1 | 1 | 4 | 4 | 1 |
| Mori et al [43] | 1 | 2 | 1 | 2 | 2 |
| Nanni et al [35] | 9 | 2 | 1 | 10 | 0 |
| Nitz et al [36] | 5 | 1 | 1 | 5 | 0 |
| Pizzutti et al [44] | 1 | 1 | 1 | 1 | 1 |
| Puppe et al [37] | 1 | 2 | 1 | 2 | 1 |
| Sestak et al [38] | 2 | 2 | 1 | 4 | 2 |
| Siddappa et al [50] | 1 | 1 | 1 | 1 | 0 |
| Swain et al [39] | 1 | 1 | 1 | 1 | 0 |
| Szijgyarto et al [40] | 5 | 2 | 1 | 10 | 0 |
| Turner et al [41] | 11 | 1 | 4 | 14 | 3 |

^a^ The same biomarker on two different scales was considered as two biomarkers

^b^ Only endpoints which were used in interaction analyses were considered

^c^ The whole group of patients was considered as one population

^d^ If it was not the product of the three previous columns, analysis of each biomarker was not performed for all endpoints and all subgroups
